# Experiencing pain: electromagnetic waves, consciousness, and the mind

**DOI:** 10.3389/fnhum.2025.1568019

**Published:** 2025-07-24

**Authors:** Richard Ambron

**Affiliations:** Departments of Cell Biology, Anatomy, and Pathology, Vagelos College of Physicians and Surgeons, Columbia University, New York, NY, United States

**Keywords:** pain, Electromagnetic waves, anterior cingulate cortex, consciousness, amygdala, nucleus accumbens

## Abstract

Studies of nociception resulted in a theory in which the quality of pain – the suffering - arises when action potentials (APs) from the thalamus that encode information about an injury induce a long-term potentiation (LTP) at synapses on pyramidal neurons in a pain center (PC) within the anterior cingulate cortex (ACC). The LTP sensitizes transmission across the synapses via the activation of adenylate cyclase-1 (AC-1) and protein kinase A (PKA). It also generates Electromagnetic (EM) waves that now contain the information about the pain. The pain is experienced when the waves reach consciousness. Blocking the AC-1, PKA, or the waves attenuates the pain. The theory was founded on the response to a simple injury. I now discuss the role of other cortical centers involved in pain. Attention to pain is governed by circuits in the anterior insula cortex (IC); fear, which enhances the intensity of pain, involves circuits in the basal nucleus of the amygdala; and reward, which can attenuate pain, is regulated by activity in the nucleus accumbens (NucA). Evidence shows that injury-evoked APs induce LTP and the generation of EM waves in the IC, amygdala, and the NucA. Interactions between the waves from the PC with those from the amygdala or NucA can enhance or reduce pain, respectively. These findings reinforce the earlier theory that the information in the EM waves results in sensory experiences in consciousness. I now propose that the summation of the sensory experiences becomes knowledge in the mind, which is an entity distinct from the brain.

## Introduction

Recent advances in molecular neurobiology, systems neuroscience, and physics are gradually shifting attention away from philosophical arguments about the nature of consciousness and the mind toward theories based on what we have learned about the functions of the brain. The human consciousness of objects and events in the world depends on the acquisition of information by our primary senses- vision, audition, touch, pain, and olfaction. The information from each sense is encoded in action potentials (APs) that propagate along axons within dedicated networks to cortical and subcortical centers in the brain where the information from each sense is processed. The information ultimately results in an experiencing the quality of each sensation: the color of flower, the sound of a bird, or the pain from an injury. Although some of the sensory pathways have been characterized in detail, it was not clear how the information acquired by our senses results in the conscious awareness of a sensory experience. This is important because these experiences are sources of information that leads to knowledge. Some prominent contemporary views attribute the consciousness of sensory experiences to the electrical activity in the brain (e.g., Baars et al., 2021; Smart, 2022; Stoljar, 2024) whereas opposing views link consciousness to the electromagnetic (EM) fields that are generated by this electrical activity (Pockett, 2013, 2017; Varela et al., 2001; John, 2002; McFadden, 2002a; McFadden, 2002b; McFadden, 2020; Buzsáki, 2006; Das, 2018; Young et al., 2022). McFadden, for example argues that EM waves arising from the synchronous firing of APs convey information into the extra-neuronal space that generates a global brain EM field that is consciousness. In general, the idea that waves have a role in consciousness failed to gain traction and has been largely ignored (e.g., MacIver, 2022; Seth and Bayne, 2022; Smart, 2022; Stoljar, 2024).

The major obstacle in attempts to understand the neurobiological basis of consciousness was the need to correlate a specific conscious sensory perception with specific neural processes amidst all of the ongoing background activity in the brain. For this reason, pain is an excellent model because it is experienced only after an injury or inflammation or due to extreme psycho-social distress and this has enabled clinicians and neuroscientists to identify the areas in the brain that are active only when patients are in pain. A study of the suffering that arises from a direct response to an injury led to a theory in which APs that encode information about the severity of the injury activate neurons within a pain center (PC) located in the anterior cingulate cortex (ACC). The activation leads to the generation of EM waves that now contain the information about the injury: the pain is then experienced when the EM waves reach consciousness. Thus, consciousness is an entity that is spatially and functionally distinct from the brain (Ambron, 2024a). This theory is consistent with the earlier ideas of Pockett, McFadden and others mentioned above.

The theory was based on the simplest case, - the direct response to an injury. However, the ultimate intensity of the pain can be shaped by several factors and these must be considered for the theory to be correct. Consequently, the objective of this narrative review is to refine further the roles of the brain, the EM waves, and consciousness in the suffering after an injury. In particular, I will address two issues. The first is attention. While it appears that the consciousness of our surroundings unfolds in a constant stream, the steam is actually discontinuous because we can only attend to one sensation at a time. Moreover, the sensation that is perceived at each moment is the one that is deemed to be most important. Suppose you are examining a flower and your finger is pierced by a thorn. The vision of the flower is immediately superseded by painfulness from the thorn. If you then hear a loud siren, the painfulness is displaced as you pay attention to the siren. The first section of this review discusses the role of the insula cortex (IC), the frontal cortex (FC), and the PC in shifting the attention from vision to pain to the sound of the siren.

The second issue is modulation of pain via the activation of circuits in the cortex. Melzack and Casey (1968) recognized that pain can be influenced by neuronal centers in the brain that mediate mood and affect and these centers have been incorporated into the more recent Integrated Pain Network (IPN) that include centers for cognition (Ambron, 2023). Consequently, the second section focuses on how circuits in the amygdala, nucleus accumbens (NucA), and the FC, influence the activity in the PC to modulate the degree of suffering. The concluding section then uses this new data to update the former theory and discuss potential relationships between sensory experiences, consciousness and the mind.

## Results and discussion

### Nociception: sensing pain

Nociception is not pain: it consists of the neuronal pathways and processes that ultimately result in pain. Nociception begins when algogenic agents released from a lesion activate receptors on the terminals of 1st order C-type neurons located in the skin, muscles and joints. These neurons are responsible for the pain that persists beyond the initial acute pain, which is mediated by the A-delta neurons (Ambron and Sinav, 2022). The binding depolarizes the terminal and evokes APs that encode information about the severity of the lesion: the more severe the lesion, the greater the number and frequency of the APs. The APs propagate to the spinal cord where they activate dendrites on 2nd order neurons. When the lesion is sufficiently serious, the activation lowers the threshold for firing the 2nd order neurons so that responses to subsequent inputs are enhanced. This phenomenon is known as central sensitization (Latremoliere and Woolf, 2009) and it shares many of the electro-physiological and molecular events that also occur at synapses in the brain and will be discussed in detail in subsequent sections. Axons from the 2nd order neurons then convey the information via the spinothalamic tract to centers in the thalamus.

### Nociception and the thalamus

The thalamus is a highly conserved subcortical structure that consists of more than 30 neuronal nuclei. It receives information from each of our senses (except olfaction) and after processing in one or more of the nuclei, the information is distributed to sensory-specific neuronal circuits in the cortex (Groh et al., 2017; Hwang et al., 2017). Nociceptive information received from the 2nd order neurons in the spinal cord enters the thalamus, is processed, and is then conveyed to areas of the cortex via two main tracts: a lateral tract to the somatosensory cortex that alerts us to the site of the injury and a medial tract to the PC, mid-cingulate cortex, NucA, and the amygdala, Yang et al., 2011; Hwang et al., 2017). The posterior region of the ventral medial thalamic nucleus (VMpo) is particularly important because it contains a somato-topographically organized cluster of neurons that respond only to pain and temperature (Blomqvist et al., 2000). VMpo receives input from the 2nd order neurons and its neurons project to the IC, thereby providing a somatosensory nociceptive input to this area. Thus, the thalamus, via its connections to all of these centers, has an essential role in shaping the intensity of pain.

### Thalamic projections to the PC for pain

Studies from several disciplines have shown that neuronal circuits within the ACC are important for experiencing pain from a physical injury (Shyu et al., 2010; Chapin et al., 2012; Fuchs et al., 2014; Zhuo, 2014; Moon and Park, 2022; Ambron, 2023, 2024a), from viscera (Morgan et al., 2005), as well as the suffering from psychological causes (Eisenberger, 2012; Xiao and Zhang, 2018). A significant advance was the finding in humans and primates of an area within Brodmann’s 24a/b in the ACC whose function is associated specifically with experiencing pain from an injury (Shyu et al., 2010; van Heukelum et al., 2020; Ambron, 2023). This PC is located near areas in the ACC that mediate attention, pain avoidance, fear, and depression (Beckmann et al., 2009; van Heukelum et al., 2020; Ambron, 2023; Journée et al., 2023). Each of these contributes in complex ways to a specific aspect of pain.

Like other areas of the mammalian cerebral cortex, the PC consists of vertical columns containing neurons that are distributed among six lamina that parallel the surface. The large pyramidal neurons in lamina V are particularly important in the context of pain. Each pyramidal neuron has a single very long apical dendrite that ascends to lamina I and II where it branches extensively and forms a large dendritic field with dendrites from adjacent pyramidal neurons. The PC has a counterpart in mice (van Heukelum et al., 2020) and by combining the results from both primates and mice it has been possible to characterize the molecular events that occur in the Lamina V pyramidal neurons when they are activated in response to an injury.

### Long-term potentiation and synaptic sensitization

The following summarizes the essential data cited in previous publications (Ambron, 2023, 2024a,b). Axons conveying nociceptive information in the medial thalamic tract form glutamatergic excitatory synapses on the spines of the thousands of dendritic branches of the pyramidal neurons in the PC. The release of glutamate (Glu) after an injury results in the synchronous activation of the post-synaptic pyramidal dendrites with two consequences (Lindén et al., 2010; Lindén et al., 2011; Pockett, 2012; Einevoll et al., 2013; Hales and Pockett, 2014; Lee et al., 2022; Ambron, 2024b,a). First is the induction of a long-term potentiation (LTP) in the dendritic spines. Second is the appearance of both local field potential (LFP) and synchronized oscillating EM waves in the extra-neuronal space around the pyramidal neurons. Both the LTP, LFP, and the EM waves are necessary for experiencing pain (Zhuo, 2014; Kang et al., 2016; Bliss et al., 2016; Lee et al., 2022; Ambron, 2023, 2024b). Notable is that similar synchronous oscillations can be elicited experimentally by applying a Theta Burst Stimulation (TBS) protocol (Li et al., 2019).

The LTP is the primary driver for the events responsible for feeling pain because it sensitizes the transmission across the thalamo-pyramidal synapses so that even the few APs elicited by a gentle touch to the injured area will evoke a response in the dendrites. This enhancement of transmission results in allodynia and explains how pain persists for hours or days after an injury, which is necessary to maintain an awareness of the injury site to avoid additional damage. Importantly, the LTP-driven sensitization maintains the presence of the LFP and EM waves. Since the duration of the sensitization is determined by the events associated with the LTP, characterizing these events is important for understanding persistent pain.

**FIGURE 1:**
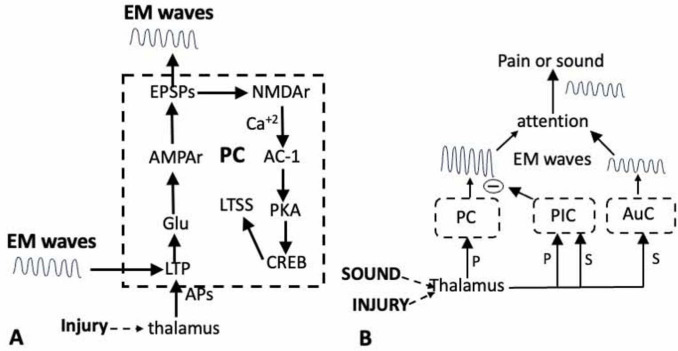
**(A)** Activation of the long-term potentiation (LTP) module in response to an injury results in long-term synaptic sensitization (LTSS), and the generation of EM waves in the PC (dashed outline). Inputs from the thalamus onto the dendrites of the pyramidal neurons in the PC induces LTP via the release of Glu. The subsequent activation of ion channels and NMDA receptors activates AC-1 and PKA resulting in the sensitization of transmission across the thalamo-pyramidal synapses and the activation of the transcription factor CREB in the pyramidal neurons. The resultant phenotypic changes sustain both the LTSS and the EM waves. The LTP can also be induced by external EM waves. **(B)**. Shifting attention from pain (P) to the sound (S) of a siren. Both stimuli first activate circuits in the thalamus and in the PC for pain and the auditory cortex (AuC) for sound, resulting in the generation of EM waves. The thalamus also activates circuits in the posterior insula cortex (PIC) whose output inhibits the circuits in the PC. This prevents the generation of the waves that would be responsible for experiencing the pain while allowing those for the sound of the siren to attain attention.

Long-term potentiation involves multiple electro-physiological and molecular events in both the presynaptic and post-synaptic terminals. For those interested in more detailed accounts, see Lee et al., 2022; Ambron, 2023, 2024b) and the many publications from the Zhuo lab. The focus here is on postsynaptic LTP, which is separated into two phases. The early phase begins when the Glu released in response to nociceptive inputs binds to α-amino-3-hydroxy-5-methyl-4-isoxazolepropionic acid (AMPA) receptors on the membrane of the post-synaptic pyramidal dendrites (Figure 1A). This results in the generation of excitatory post synaptic potentials (EPSPs) in the pyramidal dendrites. When the injury generates a sufficient number of EPSPs, it leads to the activation of *N*-methyl-D-aspartate (NMDA) receptors that have a crucial function in regulating the transmission across the synapses in response to an injury or an inflammation (Blanke and Van Dongen, 2009; Li et al., 2019; Chen et al., 2021). Its activation results in an influx of Ca^+2^ into the postsynaptic terminal that initiates the enzymatic cascades that are necessary to experience pain. Thus, the activation of the NMDA receptor directly links electro-physiological activity at the synapses to biochemical events in the dendrites.

The Ca^+2^ initiates several enzymatic reactions in the dendrites, but most important is the activation of adenylyl cyclase-1 (AC-1) that is required for the development of LTP in both mice and humans (Wei et al., 2002; Liauw et al., 2005; Liu et al., 2020; Shiers et al., 2022) and for the expression of both visceral (Zhou et al., 2021) and neuropathic pain in mice (Vadakkan et al., 2006; Shiers et al., 2022). Significantly, preventing the activation of AC-1 in the ACC does not affect basal Glu transmission or acute pain, nor does it have an effect on anxiety or fear (Wei et al., 2002; Kang et al., 2016; Li et al., 2019). Since the ACC contains centers for mood and receives inputs from the amygdala for fear (Veinante et al., 2013; Ambron, 2023) these findings are consistent with the idea that AC-1 activity is specifically associated with experiencing pain.

Adenylate cyclase-1 activates Protein Kinase A (PKA) that has a role in both phases of LTP, but its translocation into the nucleus of the pyramidal neurons in the late phase is most important. The PKA, in conjunction with other kinases, activates the cAMP Responsive Element-Binding protein (CREB; Toyoda et al., 2010; Kandel, 2012; Li et al., 2019). CREB is a transcription factor that regulates the synthesis of proteins that alter the phenotype of the pyramidal neurons thereby maintaining the LTP, the sensitization of the synapses and the generation of the LFP and EM waves (Figure 1A). These phenotypic changes are not readily reversed and when present, are responsible for strengthening that pathway.

The projections of the lamina V pyramidal neurons are the primary means by which information flows throughout the brain. It is important therefore that the sequence of biochemical events that occur in PC pyramidal neurons after an injury comprise a module (Figure 1A) that will not only strengthen the pain pathway relative to competing pathways, but will also generate EM waves (Redmond et al., 2002; Kandel, 2012; Ortega-Martínez, 2015). The output is dependent upon interactions between the pyramidal neurons and inhibitory and interneurons within the circuit that are important and are only beginning to be characterized (e.g., Swanson and Maffei, 2019). Nevertheless, this module is present in other areas of the cortex involved in pain and its activation by other inputs will reinforce that pathway and also produce EM waves.

### The insula cortex and focused attention

Pain is but one of the sensations that occurs along a discontinuous stream of consciousness. Which sensation we experience at any given moment depends on attention and this involves circuits in the IC (Starr et al., 2009; Uddin et al., 2017; Nomi et al., 2018). It has been long known that connections of the IC with the thalamus, prefrontal cortex (PFC), ACC, and amygdala, allow nociceptive information to be integrated with information related to affect, attention and decision-making (e.g., Mesulam and Mufson, 1982). Attention is a prerequisite for consciousness and from a mechanistic viewpoint it can be divided into processes that enable us to willfully focus on a stimulus and those that mediate rapid shifts in attention in response to an unexpected stimulus (see Nani et al., 2019 for a detailed discussion). Both are driven by cognitive centers in the cortex and both involve inputs from the thalamus.

Thalamic nuclei that receive information from the somatosensory system process the information from each sense and then delivers it to relevant areas of the cortex. As such, the thalamus is perfectly suited to coordinate the expression of sensory information. Consider the rapid shift in attention from the pain due to the thorn to the sound from the siren. The painfulness results from the transmission of information from the thalamus to dendrites on the pyramidal neurons in the PC. However, the thalamus, via its VMpo nucleus, also transmits somato-topically organized nociceptive information to circuits in the anterior and posterior regions of the IC that have essential roles in attending to pain (Lu et al., 2016). Clinicians know that pain can be reduced by a distracting somatosensory modality, such as touch or music (Lee et al., 2022; Lu et al., 2016) and this can be attributed to connections between circuits in the posterior IC (P-IC) and the PC. In the situation above, the sound of the siren activated neurons in the P-IC which blocked the pain by inhibiting the circuits in the PC (Figure 1B). This is an example of bottom-up modulation in which the P-IC is an interface for the interaction between pain and other incoming sensory modalities.

Pain is not always an automatic response to an injury and the response can depend on its importance relative to competing sensations. The appropriate response is often based on cognitive factors, including present context and past circumstances and the ultimate intensity of the pain can be altered by emotions such as fear. This occurs via top-down processes in which the IC receives and integrates sensory, visceral, emotional and higher-order cognitive functions that guide attention toward the most appropriate and relevant stimulus (Lu et al., 2016; Lee et al., 2022; Nomi et al., 2018; Molnar-Szakacs and Uddin, 2022).

Top-down mechanisms are complex, because they require the participation of circuits in the PFC where their roles are difficult to define. A central executive network (CEN) comprised of the dorsolateral prefrontal cortex (DL-PFC) and the anterior IC (A-IC) exhibits increased activation during stimulus-driven cognitive or affective processing and the CEN is believed to enable us to plan, willfully switch attention, and even change goals (Sridharan et al., 2008; Molnar-Szakacs and Uddin, 2022). Studies of patients with DL-PFC lesions show that the information that enters this area determines which stimulus is to receive attention, which is then implemented by its connections to the A-IC (Hornak et al., 2004). The CEN communicates with a salience network (SN) that consists of the Ventro-lateral (VL) PFC, A-IC, and the ACC. Pain is the most immediately salient sensation after an injury and studies by Lee et al., 2022 suggest that connections between the centers in the SN will focus attention on pain when it is deemed to be the most appropriate behavioral response.

At the molecular level, pyramidal neurons in the IC are activated by both visceral and neuropathic pain that involves enhanced presynaptic release of glutamate, and the postsynaptic recruitment of AMPAR and NMDA receptors resulting in the induction of LTP (Qiu et al., 2014, 2013; Lu et al., 2016; Zhuo, 2016; Bai et al., 2019; Lee et al., 2023). Both insular lesions and inhibitors of IC glutamatergic transmission have analgesic effects in neuropathic and inflammatory pain models. As in the PC, the LTP module involves the activation of AC-1, PKA and CREB and Theta burst activation of the A-IC induces LTP and the generate EM waves from the IC.

In summary, the IC is a direct interface for the interaction between pain and other sensory modalities that are vying for attention, as well as for receiving inputs from cognitive decision-making centers that impart salience. Priority should be given to identifying the inputs to the circuits in the VL-PFC and DL-PFC since these provide the information that is used to decide which sensation is to receive attention (see section “Discussion”).

### The modulation of pain: fear and the amygdala

Algophobia, a fear of pain, arises from the retrieval of a memory of an earlier painful or traumatic event. A needle, for example, will evoke fear if it had caused pain earlier in life and this is important because fear enhances the intensity of pain. Neuronal circuits involved in the fear response are located in the central nucleus (Ce) and basolateral area (BLA) of the amygdala (Pare and Duvarci, 2012; LeDoux, 2014; Arruda-Carvalho and Clem, 2015; Keifer et al., 2015; Neugebauer, 2012). Important to note is that stimulating the amygdala does not elicit pain, which indicates that the fear effect is not due to a direct connection to the PC.

Animals exposed to a threatening stimulus exhibit a freezing behavior that is a convenient measure of fear (Meulders, 2020). The behavior can be learned using a conditioning protocol by which an animal learns to associate an innocuous auditory stimulus with a noxious stimulus, such as a shock. The animal then freezes when exposed to the sound, which means that it has learned, and has a memory of the relationship between the two stimuli. During fear conditioning, information in the auditory stimulus reaches the Lateral (LA) area of the BLA via efferents from both the thalamus and the cortex. Activity-dependent changes in synaptic efficacy are key cellular mechanism for the formation and storage of memories and both Kim and Cho (2017) and Abatis et al. (2024) showed that fear learning induced LTP in a population of LA-BLA neurons. Huang and Kandel (2007) previously showed that low frequency stimulation (LFS) of a pathway from the cortex to the LA-BLA, but not that from the thalamus, elicited a PKA and protein synthesis-dependent late-phase LTP and a LFP in the LA-BLA. As in the PC pyramidal neurons, the LTP could be induced by a theta stimulation protocol and it required the activation of the NMDA receptor. By strengthening the connection between the cortex and circuits in the LA-BLA, the LTP would favor auditory information flowing from the auditory cortex for the memory of fear and would also generate EM waves from the LA-BLA.

The brief exposure to the sound elicits the fear response, but also initiates a re-consolidation process that maintains the original memory. Mamiya et al. (2009) found that this process involved activation of CREB-mediated gene expression in the amygdala and the hippocampus, which is where the memory would be preserved. In addition, both Karalis et al. (2016) and Davis et al. (2017) reported that the fear conditioning elicited synchronized oscillations from the BLA neurons that would generate EM waves, which is consistent with the findings by Abatis et al. (2024) and Huang and Kandel (2007) mentioned above. However, the fear response is also adaptive and continued exposure to the auditory stimulus attenuates the response via inputs from the medial PF cortex to neurons in the LA-BLA. Thus, once again, decisions imposed from circuits in the PF cortex have major behavioral consequences (Arruda-Carvalho and Clem, 2015). The PF cortex might have an even more important role because there is evidence that, in addition to the hippocampus, circuits in the PF cortex are necessary for the storage of memories associated with fear (Lee et al., 2022).

The freezing behavior is accompanied by changes in heart rate and other autonomic effects due to efferents from the BLA to the hypothalamus. Interestingly, fear in mice also occurs due to an innate response to the urine from a predator that does not elicit the autonomic responses. The responses involve different pathways between lamina V Pyramidal neurons in the BLA and circuits in the ACC (Jhang et al., 2018): those to the caudal ACC control the innate fear response, while those to the rostral ACC control the learned fear. Becker et al. (2023) also found connections between BLA neurons and those in the ACC, specifically within area 24a/b, which contains the PC. Stimulation of this pathway elicits depressive-like symptoms in the absence of pain (see also Sellmeijer et al., 2018). Inhibiting the pathway blocked the expression of the depressive consequences of chronic pain, without affecting anxiety, aversiveness, or mechanical hypersensitivity. Thus, in addition to its role in directly experiencing pain, the ACC participates in both the fear response and the comorbidity of chronic pain and depression that can modulate the pain.

The Ce neurons in the amygdala have two roles in pain (Neugebauer, 2012). Inputs from the lateral parabrachial (PB) area in the brainstem convey nociceptive information about an injury, as well as visceral information due to colorectal distention and cystitis. How these influence pain is not clear. Other Ce neurons appear to be directly involved in fear learning because neurons in the anterior Ce exhibit synaptic plasticity in response to fear conditioning that involves NMDA receptors and CREB expression (Pape and Pare, 2010; Pare and Duvarci, 2012). The activation of the LTP module means that the A-Ce undergoes persistent functional changes in response to fear conditioning.

In conclusion, there are several neuronal networks in the BLA and Ce that contribute to storing and expressing fear memories. Inputs from the areas in the PF cortex, thalamus and IC to the BLA contain information that has already been processed by these centers, whereas the Ce obtains direct nociceptive information from the parabrachial nucleus (PBN). All of these will influence the experiencing of pain. There are several inputs to the ACC from the amygdala, yet none of these pathways can explain how fear enhances pain.

### The modulation of pain: reward and belief

The presence of ongoing pain in animals provides a motivation to seek relief that is a measure of pain aversiveness. In other words, relief from pain is rewarding and it reinforces behaviors that result in avoidance of pain. The NucA is important as a key brain region mediating a variety of behaviors, including reward and satisfaction and it receives direct input in the form of glutamatergic projections from the amygdala, hippocampus, thalamus, ACC, and PFC (see Salgado and Kaplitt, 2015 for a comprehensive review), indicating that the NucA activity will have an effect on pain (Porreca and Navratilova, 2017; Harris and Peng, 2020).

Single unit recordings in animals and fMRI in humans have demonstrated increases in NucA activity following a reward that was associated with EM waves (Cohen et al., 2007). Connections between the amygdala and the NucA in the response to pain are particularly important. Namburi et al. (2015) compared two populations of BLA neurons: those that project to the NucA region associated with reward-like behavior and those that project to neurons in the Ce that are associated with conditioned fear. Mice underwent either fear conditioning or a reward training protocol after which the strength of the synaptic input received by each population of BLA neurons was examined. Fear conditioning strengthened the synapses onto the Ce-projecting neurons but reduced the strength of synapses onto NucA-projecting neurons. Reward conditioning had the opposite effect: it increased the strength of synapses on the NucA projecting neurons but reduced in it in the CeM projecting neurons. These results suggest that stimulating a reward pathway will reduce pain. Significant in this regard is the report from Cohen et al. (2007) who detected gamma (40–80 Hz) and alpha (8–12 Hz) oscillations in the human NucA. They found that a reward elicited increased gamma activity bursts immediately following alpha troughs, implying that the slower oscillations control the timing of different neural networks that represent different kinds of information.

Among the most influential effects on pain are responses to beliefs (Ambron and Sinav, 2022). A good example is the placebo effect where pain is attenuated in response to a treatment that has no therapeutic value. fMRI imaging of patients exhibiting a successful placebo consistently detected increased activity in the PFC, NucA, and the periaqueductal gray (PAG) and reduced activity in the thalamus, ACC, somatosensory cortex, amygdala, A-IC, and the spinal cord (Wager et al., 2013; Wager and Atlas, 2015; Ambron and Sinav, 2022). Activation of the PAG will reduce pain via the endogenous opioid system (Ambron and Sinav, 2022) and the PFC is involved because the effect requires a decision or belief that the treatment will be effective. These results are exactly what would be predicted given what we know about the functions of these centers in nociception. Evidence indicates that it is the activation of dopaminergic receptors in the NucA via inputs from the rostral ACC that mediate the pain relief from the placebo (Johansen and Fields, 2004; Qu et al., 2011) and this important connection warrants further investigation given its potential importance for managing pain (Porreca and Navratilova, 2017).

**FIGURE 2:**
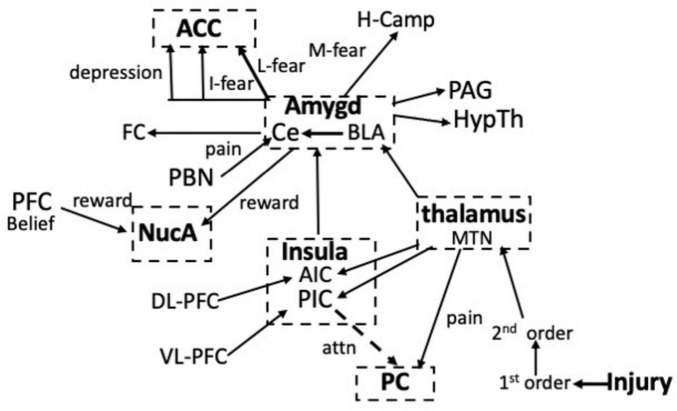
Interactions between the neuronal centers involved in pain, attention, fear, and reward in response to an injury. Information about the pain enters the network via projections from the median thalamic nuclei (MTN) to the PC or from the parabrachial nucleus (PBN) to the amygdala. Learned (L), Innate (I) and the memory (M) of fear via the hippocampus (H-Camp), as well as fear conditioning (FC) result from the activation of circuits in the amygdala. Reward involves circuits in the nucleus accumbens (NucA). Note that the PC and the ACC are separated in the schematic. See text for details.

Figure 2 maps the various communications between the neuronal centers involved in pain and updates the earlier IPN. The map is by no means complete and will be expanded as more is learned about the role of the frontal cortex. Nevertheless, if we exclude psychological-social causes of pain, the map shows that both somatic and visceral pain enter the network at only two sites; via the somatosensory system or the parabrachial pathway. All of the other centers regulate attention via the inputs from the dorsal and ventral PFC, or modulate the pain in response to fear or reward. These function together to ensure that the ultimate experiencing of the pain is the best behavioral response to a painful incident. Inputs into these centers that activate the LTP module will generate EM waves.

Chronic pain is a major clinical problem and characterizing the various circuits that contribute to pain might provide new targets for the development of novel analgesics. Significant is that communications between all the centers in the network are mediated by APs: nowhere is there an explanation as to how these activities can explain suffering. Moreover, inhibiting the activation of the NMDA receptor, AC-1, or PKA in the ACC prevented the experience of pain. Thus, they are necessary for pain, but also provide no clear path for understanding how pain is experienced because they are not unique to nociception but are also activated in neuronal systems with other functions. Nevertheless, these components are important because they lead to the activation of CREB and the subsequent synthesis of the proteins that alter the phenotype of the pyramidal neurons. The alterations will strengthen the nociceptive pathway relative to others and will maintain the allodynia until the lesion is healed. The other consequence of the inhibition was to prevent the appearance of the EM waves in the space around the pyramidal neurons and what follows below is evidence that they have an essential role in experiencing pain.

### Properties of synchronized oscillating waves and the distribution of nociceptive sensory information

The oscillating LFP is generated by synchronized synaptic ionic currents arising from the large array of pyramidal neuron dendrites (e.g., Buzsáki et al., 2012; Destexhe and Bedard, 2013). The LFP can alter the activity of circuits nearby via ephaptic effects (Anastassiou and Koch, 2015) but its influence is thought to be limited by distance (Buzsáki et al., 2012), which in the visual system was approximately 400 um (Dubey and Ray, 2016). However, the waves emanating from the thousands of pyramidal neurons could extend much further and this is important because we know from the data above that the ACC contains circuits for attention, depression, and fear. All of which could potentially be regulated by the LFP from the PC. Experiments to determine the role of the LFP in mice have been described in detail elsewhere (Ambron, 2023).

The LFP originates in two interconnected fundamental physical fields of nervous tissue: an electric field, and a magnetic field (Hales and Pockett, 2014) and the resultant synchronized oscillating EM waves can travel great distances and are detected at the scalp by electroencephalography (EEG) and magnetoencephalography (MEG). Even the magnetic waves generated in the motor cortex by a simple hand movement were detected above the head (Brazdzionis et al., 2022a,b) so it would be expected that the much more powerful waves from the PC can communicate with both cortical and subcortical centers far from the PC. Moreover, the EM waves from the PC will be present as long as the synaptic sensitization persists, which can be a very long time since the sensitization is maintained by the phenotypic changes discussed above.

Electromagnetic waves have two properties that make them especially suited for nociception. First, they contain packets of energy called photons that can directly influence the activity of neurons in the cortex (Richardson et al., 1984; Fröhlich, 2014; Ye et al., 2022). For example, repetitive transcranial magnetic stimulation (rTMS) protocols have shown that theta burst protocols can either induce or inhibit LTP in the cortex depending on their frequency (Vlachos et al., 2012; Komaki et al., 2014; Lenz et al., 2015; Rajan et al., 2017) which strongly suggests that the EM waves from the PC could regulate LTP modules in distant areas of the cortex (Figure 1A). Perhaps more telling is that TMS is already being used to deliver external EM waves to attenuate pain (Chail et al., 2018; Paolucci et al., 2020; Fernandes et al., 2022; Kim et al., 2022) and that deep brain stimulation at frequencies up to 20 Hz successfully provided pain relief in patients with chronic phantom limb pain by suppressing the enhanced activity in the ACC (Mohseni et al., 2012). These studies clearly show that internally generated and externally applied EM waves have multiple influences on the functions of circuits in the brain and dispel the idea that the waves are mere artifacts or epiphenomena without purpose.

The other important property of EM waves is that they contain information about their source that is encoded in their frequency, amplitude and phase (e.g., Tentrup et al., 2017; Brazdzionis et al., 2022a,b). Thus, the information about the intensity of the pain from an injury that was originally contained in the APs propagating through the nociceptive network is now encoded in the synchronized, oscillating EM waves where it can be widely disseminated throughout the brain (de Haan et al., 2020; Brazdzionis et al., 2022a,b). Consequently, it has been proposed that EM waves are a means by which information is rapidly exchanged throughout the brain (Varela et al., 2001; Schnitzler and Gross, 2005; Fröhlich, 2014; Fröhlich and McCormick, 2013; Ploner et al., 2017; Ambron, 2024b).

**FIGURE 3:**
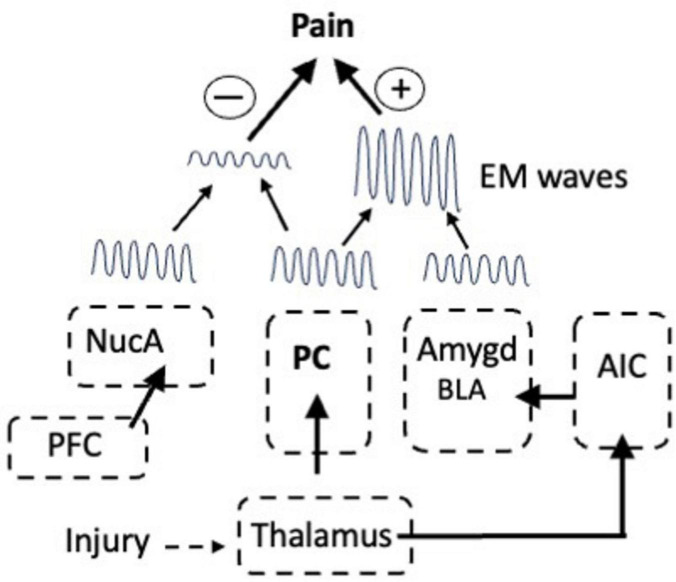
The modulation of pain via interactions between EM waves emitted from the nucleus accumbens (NucA) for reward, the PC for pain, and in response to inputs from the AIC, from the Amygdala for fear. In-phase waves from the PC and amygdala summate to increase pain in response to fear whereas out-of-phase waves from the NucA and PC interfere with one another, thereby reducing the pain. prefrontal cortex (PFC).

That EM waves contain information is significant because in addition to the waves from the PC, pain-associated EM waves in the high theta (6–9 Hz) and low β frequencies (12–16 Hz) are emitted from the IC, NucA, and amygdala (Stern et al., 2005; Stern et al., 2005; Ploner et al., 2017; Lee et al., 2022). The waves from each center are created by the activation of the corresponding LTP module and they contain information that will influence the intensity of the pain, yet they are widely separated in the brain: how then is the information assembled into a single consciousness of pain? This is known as the combination problem and the waves are a potential solution (Ambron, 2023, 2024a). Fear arises due to the activation of circuits in the amygdala and fear exacerbates pain, but stimulating the amygdala does not evoke pain and all of the axonal connections between the amygdala and the ACC mediate other functions. Davis et al. (2017) showed that fear-associated oscillations in the low theta range (3–6 Hz) are emitted from the amygdala and we can therefore ask what happens when these interact with the theta oscillating waves from the PC? The outcome is governed by superposition, a fundamental concept in wave mechanics. When waves with the same frequency and phase merge, the amplitude of the wave that results is the algebraic sum of the amplitude of the two waves. Consequently, a theta wave from the PC with the same frequency and phase as that from the amygdala would add to the PC wave and the intensity of the pain would increase (Figure 3).

The theta waves from the amygdala in mice are also important for the memory of fear (Schönfeld and Wojtecki, 2019). Thus, the retrieval of fearful memories leads to theta oscillatory coherence between the BLA and the hippocampus (Seidenbecher et al., 2003). Increased communication with the BLA would enhance processing of fearful memories as opposed to more neutral ones. Cervera-Ferri et al. (2016) reported that Deep Brain Stimulation of the cingulate gyrus increased oscillatory activity in the BLA and enhanced coherence between the BLA and the hippocampus. This would provide a way for pain to enhance a memory of a fearful event.

On the other hand, when two waves are completely out of phase the waves will either cancel each other or the wave with the greater amplitude will emerge, but with reduced amplitude. Even when two waves of different frequencies interact, such that the peak of one wave aligns with a trough of the other, it will cause destructive interference and the cancellation of the wave. Mice can learn to ignore a fearful stimulus, which is known as fear extinction, and Davis et al. (2017) showed that interneurons in the BLA have a pivotal role in fear extinction learning in mice because they generate oscillations in the alpha range (6–12 Hz) that interfere with fear-associated oscillations in the low theta range (3–6 Hz). Thus, there are many ways in which EM waves from multiple sources can interact and waves from the NucA for reward could cancel or reduce the waves from the PC thereby attenuating the intensity of the pain (Figure 3).

Pain is often considered to be subjective, which is correct in that the same noxious stimulus would elicit a different intensity of pain among individuals and even in the same person at different times. However, it is not subjective, meaning that it is random. The data above clearly show that the variability in the responses to an injury are not random but can be explained by the selective activation of specific circuits in the brain that collectively utilize the information about an injury to maximize the chances of an appropriate behavioral response.

## Summary and conclusion

Earlier studies (Ambron, 2023, 2024b) provided evidence that the APs encoding information about the severity of a simple injury activate pyramidal neurons in the PC resulting in the transformation of the information into EM waves. This led to a theory in which painfulness arises when nociceptive information in the EM waves reaches consciousness. To experience pain, therefore requires the participation of both the physical brain and non-material consciousness and that the two communicate via EM waves (Ambron, 2024a). The data presented above support this theory and provide additional evidence as to the importance of EM waves in understanding the consciousness of pain.

The degree of suffering after an injury or inflammation is regulated by interactions between a relatively limited number of cortical and sub-cortical circuits. Thus, the intensity of pain is increased by fear, due to the activation of circuits in the amygdala, whereas painfulness is attenuated by reward, which is due to the activation of circuits in the NucA (Figures 2, 3). In a successful placebo effect, activity was increased in the PFC, the NucA, and the PAG and was reduced in the thalamus, ACC, somatosensory cortex, amygdala, A-IC, which is exactly what would be predicted given their functions. In addition, the LTP modules involved in processing pain information are activated only when they are needed to elicit and reinforce an appropriate response: at other times they are silent. Nevertheless, the activation alone cannot account for the painfulness and the data show that the EM waves emitted from the centers that are activated are also necessary. We therefore have two routes by which nociceptive information is communicated. First is via APs propagating along axonal tracts and the second is by the EM waves that are emitted into the extra-neuronal space from the PC, IC, amygdala, and NucA. Both routes integrate information, but at very different time scales: APs exchange information orders of magnitude slower than that of the EM waves. One way they could work together, however, would be if the function of the APs is to activate the circuits that provide the optimal response to the injury, for example those in the PC and the LA-BLA. Each activated circuit then generates EM waves that rapidly integrate and assemble the information with the waves from other centers and then send it to be processed in consciousness. In other words, the relationship between anatomical pathways and oscillating EM waves allows the processing of sensory information to be carried out almost simultaneously at multiple temporal and spatial scales and over great distances.

**FIGURE 4:**
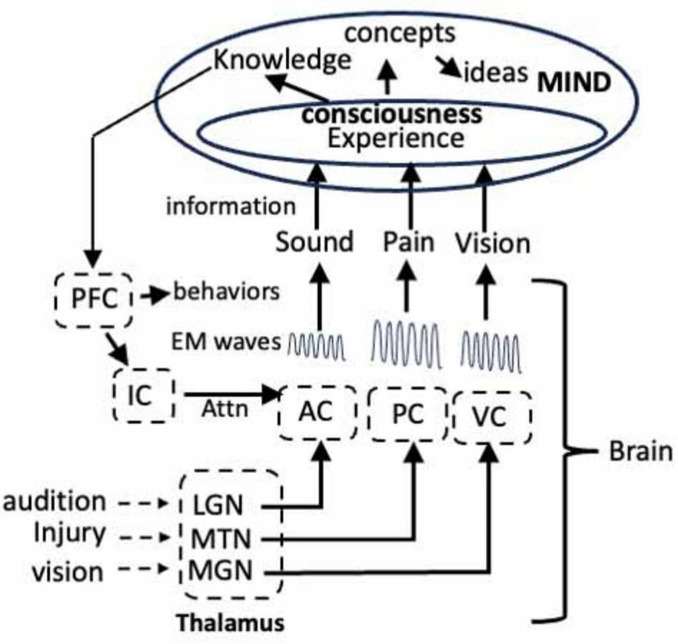
Model showing the flow of information from the auditory cortex (AC), visual cortex (VC), and pain through the thalamus to the corresponding centers in the cortex where the information encoding each sensory experience is transformed into unique EM waves. The waves enter consciousness in the mind where the sensory experiences achieve consciousness. The information becomes knowledge in the mind and the knowledge is communicated via EM waves to circuits in the frontal cortex where they induce LTP. The LTP alters the activity of the neurons in the circuit that directs attention, via the IC, and initiate behaviors. LGN, lateral geniculate nucleus; MGN, medial geniculate nucleus; MTN, medial thalamic nuclei. See text for details.

The studies of pain have provided important insights as to how sensory information culminates in a consciousness of an injury. Since audition, vision and the activation of the IC also generate unique EM waves, we can extrapolate the data from the studies of pain to create a general theory of mind (Figure 4). Thus, at every waking instant in time, we attend to one of these senses, thereby resulting in the generation of EM waves that encode information unique to that sensation. After enhancement or attenuation via interactions with other waves, the information in each of the waves becomes a sensory experience in consciousness and it is the summation of all these experiences in consciousness that culminates in a coherent conceptualization of the world over time. Each conscious sensory experience also yields information that is conveyed to the mind where it becomes the knowledge that is the foundation for ideas, etc. That is, the brain collects and collates information from our senses, converts the information into EM waves that then convey the information to consciousness, which communicates with the mind. What cannot be excluded is the intriguing possibility that the brain-mind interaction might be a closed loop in which the mind influences the activity of neurons in the brain via ephaptic interactions (MacIver, 2022).

This is only a working model that can be used to guide future studies. The nature of the mind - what it is, where it is - and its relationship to consciousness has occupied prominent philosophers, theologians, and naturalists from ancient Greece to the present yet there is no unequivocal understanding of what is meant by the mind or consciousness. Nevertheless, the theory is useful because it views both consciousness and the mind as entities with defined functions. It also shows that a plausible empirical approach to understanding both is to study the EM waves because the information in the waves is necessary for both functions. Critical is that the model is based on hypotheses that can be rigorously tested by delivering EM waves via rTMS to alter brain activity. Moreover, these protocols are non-invasive and can be applied to humans. The key is to determine the frequency of the EM waves involved in experiencing pain. Animal models might be useful here since optogenetic protocols can be devised to activate the pyramidal neurons. Activation should result in pain and the generation of EM waves. The frequency of the waves can then be determined by using TMS to apply a spectrum of EM waves to identify the frequency of the wave that cancels the pain. In other words, block the waves, block the pain.

In conclusion, the data presented in this review, and in the previous publications, represent a radical departure from traditional views of the brain because they indicate that sensory experiences, consciousness, knowledge, and the higher mental faculties that were formerly considered functions of the brain are now attributed to consciousness and the mind.

## Data Availability

Publicly available datasets were analyzed in this study. This data can be found here: My article presents a theory of consciousness that is based on information from the literature that is listed in the references section.
